# Comparison of predicting cardiovascular disease hospitalization using individual, ZIP code-derived, and machine learning model-predicted educational attainment in New York City

**DOI:** 10.1371/journal.pone.0297919

**Published:** 2024-02-08

**Authors:** Kullaya Takkavatakarn, Yang Dai, Huei Hsun Wen, Justin Kauffman, Alexander Charney, Steven G. Coca, Girish N. Nadkarni, Lili Chan

**Affiliations:** 1 Division of Nephrology, Department of Medicine, Icahn School of Medicine at Mount Sinai, New York, NY, United States of America; 2 Division of Nephrology, Department of Medicine, King Chulalongkorn Memorial Hospital, Chulalongkorn University, Bangkok, Thailand; 3 The Charles Bronfman Institute for Personalized Medicine, Icahn School of Medicine at Mount Sinai, New York, NY, United States of America; 4 Division of Data Driven and Digital Medicine, Icahn School of Medicine at Mount Sinai, New York, NY, United States of America; New York University, UNITED STATES

## Abstract

**Background:**

Area-level social determinants of health (SDOH) based on patients’ ZIP codes or census tracts have been commonly used in research instead of individual SDOHs. To our knowledge, whether machine learning (ML) could be used to derive individual SDOH measures, specifically individual educational attainment, is unknown.

**Methods:**

This is a retrospective study using data from the Mount Sinai Bio*Me* Biobank. We included participants that completed a validated questionnaire on educational attainment and had home addresses in New York City. ZIP code-level education was derived from the American Community Survey matched for the participant’s gender and race/ethnicity. We tested several algorithms to predict individual educational attainment from routinely collected clinical and demographic data. To evaluate how using different measures of educational attainment will impact model performance, we developed three distinct models for predicting cardiovascular (CVD) hospitalization. Educational attainment was imputed into models as either survey-derived, ZIP code-derived, or ML-predicted educational attainment.

**Results:**

A total of 20,805 participants met inclusion criteria. Concordance between survey and ZIP code-derived education was 47%, while the concordance between survey and ML model-predicted education was 67%. A total of 13,715 patients from the cohort were included into our CVD hospitalization prediction models, of which 1,538 (11.2%) had a history of CVD hospitalization. The AUROC of the model predicting CVD hospitalization using survey-derived education was significantly higher than the model using ZIP code-level education (0.77 versus 0.72; p < 0.001) and the model using ML model-predicted education (0.77 versus 0.75; p < 0.001). The AUROC for the model using ML model-predicted education was also significantly higher than that using ZIP code-level education (p = 0.003).

**Conclusion:**

The concordance of survey and ZIP code-level educational attainment in NYC was low. As expected, the model utilizing survey-derived education achieved the highest performance. The model incorporating our ML model-predicted education outperformed the model relying on ZIP code-derived education. Implementing ML techniques can improve the accuracy of SDOH data and consequently increase the predictive performance of outcome models.

## Introduction

Social determinants of health (SDOH) are non-medical factors that influence health outcomes. The World Health Organization defined SDOH as the conditions in which people are born, grow, live, work, age, and the wider set of forces and systems shaping the conditions of daily life. Numerous studies have highlighted the important role of SDOH, such as educational attainment, as crucial determinants of health outcomes [1–3]. Despite the significance of SDOH, acquiring individual SDOH information is challenging. Individual SDOH is frequently absent from electronic health records (EHRs) or documented as free text which is not available for statistical analysis. Surveys, which are the standard for obtaining individual SDOH require substantial time and resources. Therefore, in lieu of individual SDOHs, researchers have commonly utilized area-level SDOHs based on patients’ geographic location, such as ZIP codes [4].

Prior studies evaluating the impact of including SDOH into predictive model performance have resulted in mixed results [5–11]. Hammond et al. [5] demonstrated that adjusting for SDOH improved the accuracy of cardiovascular hospitalization and mortality regression prediction models. Segar et al. [7] also illustrated that the addition of SDOH measures improved the performance of ML-based random forest algorithms to predict heart failure mortality, but only in Black patients. In contrast, Bhavsar et al. [9] showed that although neighborhood socioeconomic status (SES) was associated with health outcomes, SES did not improve the risk prediction of clinical events, including emergency department visits, outpatient visits, and hospitalizations, above and beyond what is already provided by the EHR data. A systematic review of 13 studies using SDOH for risk prediction revealed that eight of them demonstrated a significant improvement in predictive models when incorporating SDOH measures [11]. Notably, all studies that integrated individual-level SDOH data reported a significant enhancement in performance. In contrast, among the six studies that merged neighborhood-level SDOH data with EHR data, only one showed a significant improvement [11]. At present, to our knowledge, there is no study directly comparing the impact of using individual and neighborhood SDOH measures in predictive risk models.

We hypothesized that area-level SDOH measures may not accurately represent individual SDOHs, especially in highly diverse urban neighborhoods, and this discordance will negatively impact performance of health outcome prediction models resulting in the above conflicting results. To develop a method for predicting individual level education using features readily available in the EHR, we employed machine learning (ML). ML which uses more sophisticated mathematical functions than traditional statistics will typically yield superior performance when predicting outcomes influenced by a large number of variables with nonlinear and complex interactions [12, 13] and therefore improve accuracy of individual SDOH estimates. To address these gaps in the literature, first, we assessed the concordance between ZIP code-level educational attainment and survey-derived data using survey-derived data as a gold standard. We specifically focused on educational attainment as a significant available SDOH data. We then utilized ML models to derive educational achievement using routinely collected clinical and sociodemographic factors. To evaluate the impact of SDOH on the clinical outcome prediction, we focused on cardiovascular disease (CVD) hospitalization. The incidence of CVD is clearly associated with lifestyle behaviors, including smoking, limited exercise, obesity, and diet [14, 15]. Higher education may reduce the risk of CVD due to increased health knowledge and awareness, leading to the improvement of lifestyle behaviors [16]. Higher education may also be associated with stronger social connections and access to health care [17, 18]. Several previous studies have established the association of education and CVD outcomes [19–21]. Therefore, we subsequently conducted a comparative analysis of the performance of three distinct predictive models for CVD hospitalization.

## Methods

### Study population and data sources

We obtained individual data from the Bio*Me* Biobank, which is an ongoing, non-selective patient-based EHR-linked biorepository at the Mount Sinai Health System (New York, NY, USA) [22]. Participants were recruited into the biobank since September 2007. At enrollment, participants completed a questionnaire which included questions on age, gender, race/ethnicity, and educational attainment (S1 Questionnaire). The Bio*Me* Biobank received ethics approval from the Institutional Review Board of the Mount Sinai School of Medicine (STUDY-11-01139). All participants provided written informed consent for the study. As we focused on educational attainment, we included only participants who were at least 25 years and completed the questionnaire from 2007 through 2020. We excluded individuals with missing data for gender, race/ethnicity, educational attainment, and NYC ZIP code home address.

We used data from the American Community Survey (ACS) to derive area-level education [23]. ACS provides estimates of the characteristics of the population such as education, income, employment, detailed race and ethnicity, and health insurance coverage. Estimates from the ACS serve as a complement to the population data collected by the U.S. Census Bureau. Continuous data collection throughout the year is used to produce estimates for each calendar year. These estimations are founded on data collected over time, as opposed to a single point in time. The ACS 5-year estimates are compiled from aggregated data spanning a 5-year period, allowing for comprehensive SDOH attribute estimates at the block, census tract, ZIP code, and county levels. These estimates are available as 5-year aggregate files from 2007 to 2020.

We chose the ZIP Code Tabulation Area (ZCTA) as the spatial scale for analysis to estimate area variations at the neighborhood level. ZCTAs are generalized areal representations of United States Postal Service (USPS) ZIP code service areas. To achieve ZIP code-level educational attainment, we linked individual participants in the Bio*Me* Biobank to ACS 5-year estimates of educational attainment according to the participant’s survey completion year. This linkage was based on the participant’s ZIP code, gender, and race/ethnicity.

### Measures

#### Race/Ethnicity and educational attainment

Participant’s race/ethnicity was categorized into seven groups: American Indian/Alaska Native, Asian, Black/African American, Hispanic/Latino, Native Hawaiian/Other Pacific Islander, White, and others.

We defined educational attainment as the highest level of education obtained and was categorized into four groups: less than a high school diploma, high school graduate, some college or associate degree, and bachelor’s degree or higher.

#### Health outcome

The primary outcome was CVD hospitalization within 5 years after enrollment in the Bio*Me* biobank. Individual CVD hospitalizations were identified using the Clinical Classification Software Refined (CCSR) to aggregate the diagnosis codes from the International Classification of Diseases, 10th Revision, Clinical Modification (ICD-10-CM) [24] recorded in the Mount Sinai Health System. CVD hospitalization includes the primary diagnosis code in the category of ’Diseases of the circulatory system’.

#### Individual education prediction model development

For feature selection, we collected data on patient demographics (age, sex, race/ethnicity), comorbidities derived from the ICD-10 codes according to the Elixhauser comorbidity index, the history of tobacco, alcohol, drug use, and ZIP code-level educational attainment. The Elixhauser comorbidity index measures a patient’s comorbidity based on ICD codes, which are weighted based on the association of each comorbidity with death, resulting in a concise summary index of comorbidity [25, 26]. Based on the patient’s ZCTA, we also included the dissimilarity index, which measures the segregation of Blacks from Whites, and the Gini index, which measures income inequality.

We then developed three models to predict educational attainment.

- Model 1 utilized only ZIP code-level education.
- Model 2 included ZIP code-level education + demographic variables and comorbidity + use of tobacco, alcohol, and drug.
- Model 3 included ZIP code-level education + demographic variables and comorbidity + use of tobacco, alcohol, and drug + other neighborhood SDOHs (GINI and dissimilarity indices).

We chose three ML algorithms that are well-suited for multiclass classification, where our outcome could be one of three or more possibilities: Naïve Bayes (NB), decision tree (DT), and random forest (RF).

NB is a probabilistic machine learning algorithm commonly used for binary and multiclass classification tasks. It is based on Bayes’ theorem and assumes that the features used for classification are conditionally independent [27].

DT is a supervised ML algorithm used for classification and regression. It has a hierarchical tree structure which consists of a root node, branches, internal nodes, and leaf nodes. This algorithm is fast, easy to use and can naturally handle multiclass classification [28].

RF is an ensemble algorithm, which combines multiple decision trees. Each tree is built independently, and the final prediction is obtained by aggregating the predictions of all trees by the maximum vote for classification problems. This reduces overfitting and increases accuracy [29, 30].

Before modeling, all categorical variables with more than two factors were converted into dummy variables. Subsequently, the dataset was randomly divided into training and test sets, with a 75/25 split. We employed five-fold cross-validation for all our ML models. We conducted subgroup analysis by evaluating performance of our models in different race/ethnicity groups and by White- or Black-predominant ZIP codes (defined as more than 50 percent of people residing in that ZIP code identifying as that race/ethnicity).

#### CVD hospitalization prediction model development

For CVD hospitalization prediction models, we included only participants with a history of hospital visits and had ICD-10 records in the Mount Sinai Health System. The index date was recorded as the Bio*Me* biobank enrollment. We then followed patients until 5 years after the enrollment date to predict the 5-year risk of CVD hospitalization. To evaluate changes in prediction model performance based on how educational attainment is derived, we first built a model without considering educational attainment. These models utilized patient demographics (age, gender, and race/ethnicity) and the baseline Elixhauser comorbidity index score, as additional covariates to predict CVD hospitalization as a binary classification. Then we included educational attainment in three different ways: individual-level educational attainment from surveys, ZIP code-level educational attainment, and educational attainment predicted from the most accurate ML model.

#### Hyperparameter tuning

We conducted a grid search over a set of chosen parameters to find the optimal settings. Grid search is a technique used in ML to search and find the optimal combination of hyperparameters for a given model. Hyperparameters are ML parameters that are used to control the algorithm. For example, the learning rate and number of trees are hyperparameters. The Gird search technique systematically explores a predefined set of hyperparameter values, creating a grid of possible combinations. We implemented the grid search using the default setting of GridSearchCV() function from scikit-learn [31] and explored hyperparameter combinations listed in **S1 Table** to identify the configuration that optimized model performance. Model performance was evaluated using 5-fold cross-validation. The selected model was chosen based on that with the best-performing parameters.

### Statistical analysis

Categorical data are described as numbers and percentages. Continuous data are summarized as mean ± standard deviation (SD) for normally distributed variables or median (interquartile range; IQR) for non-normally distributed variables. We used Student’s T test for normally distributed continuous variables, χ2 for categorical variables, and Kruskal-Wallis for non-normally distributed continuous variables. Cohen’s κ index was used to assess the agreement between survey and ZIP code-derived education, as well as survey and ML-predicted education. To evaluate model performance, we assessed the accuracy, precision, and F1-score. The area under the receiver operating characteristic (AUROC) curve and the area under the precision-recall curve (AUPRC) are also used as metrics to measure prediction model performance. The AUROC curve of each prediction model was pairwise compared using the DeLong test. A p < 0.05 was considered statistically significant. All analyses were performed using the *scikit-learn* [31], *censusgeocode*, and *matplotlib* [32] libraries within Python 3.8.

## Results

### Baseline characteristics

We included 20,805 patients from 185 unique ZIP codes in NYC for analysis. S1 Fig presents the CONSORT diagram. The mean age of the cohort was 52.8±15.8 years. Of these patients, 12,111 (58%) patients were female, 8,858 (43%) were Hispanic, 5,250 (25%) were White, and 4,446 (22%) were Black.

**Fig 1:**
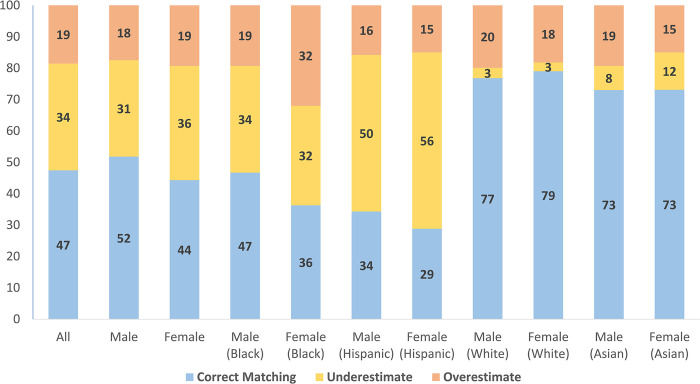
The percentage of matches between individual and ZIP code education levels by gender and race.

Regarding educational attainment according to surveys, 9% of patients had less than a high school diploma, 37% completed high school, 1% graduated with some college or associate degree, and 53% had a bachelor’s degree or higher. Based on the patient’s ZIP code, gender, and race, the educational attainment at the ZIP code level revealed that 25% had completed less than a high school diploma, 24% had completed high school, 8% had completed an associate degree, and 43% had a bachelor’s degree (Table 1). The concordance rate between survey education and ZIP code-level education was 47%. The weighted Cohen’s κ coefficients indicating the concordance between survey-derived and ZIP code-derived education was 0.32 (95% CI 0.31 to 0.34; p = 0.206). Fig 1 illustrates the percentage of matches between individual and ZIP code education levels by gender and race.

**Table 1:** Baseline characteristics of participants in cohort.

Characteristics	
Age	53 ± 16
**Female**	12,111 (58%)
**Race**	
Hispanic	8858 (43%)
White	5350 (25%)
Black	4446 (22%)
Asian	1121 (5%)
Others	1130 (5%)
**Comorbidity**	
Hypertension	8420 (40%)
DM	4260 (21%)
Coronary artery disease	2157 (10%)
Peripheral arterial disease	2176 (10%)
Arrhythmia	989 (5%)
Valvular heart disease	874 (4%)
Pulmonary vascular disease	491 (2%)
Liver disease	1869 (9%)
HIV infection	1532 (7%)
Solid malignancy	2172 (10%)
Lymphoma	251 (1%)
Anemia	2326 (11%)
Peptic ulcer	258 (1%)
Connective tissue diseases	1161 (6%)
Psychosis	687 (3%)
Depression	4865 (23%)
**Tobacco use**	
Current	4221 (20%)
Former	7220 (35%)
**Alcohol use**	
Current	9857 (47%)
Former	1070 (5%)
**Individual educational attainment**	
Less than a high school diploma	1880 (9%)
High school graduate	7642 (37%)
Some college or associate degree	237 (1%)
Bachelor’s degree or higher	11046 (53%)
**ZIP code-level educational attainment**	
Less than a high school diploma	5302 (25%)
High school graduate	4929 (24%)
Some college or associate degree	1630 (8%)
Bachelor’s degree or higher	8944 (43%)

### ML models to predict individual education

We split the data into training (n = 15,603) and test (n = 5,202) datasets. In Model 1, which included only ZIP code-level education, all algorithms showed individual education prediction accuracies ranging from 0.55 to 0.57, with AUROC values ranging from 0.64 to 0.65. In Model 2, which included ZIP code-level education, demographic variables, and comorbidity, the AUROCs improved to a range of 0.69 to 0.75. Model 3, which included ZIP code-level education, demographic data, comorbidity, GINI, and dissimilarity indices, gave the best performance. RF revealed the highest AUROC (0.77 (0.76 to 0.78)) (Table 2) of the models in predicting individual education. The concordance rate between RF model-predicted education and survey education was 67%. The weighted Cohen’s κ coefficients indicating the concordance between survey-derived and RF model-predicted education was 0.45.

**Table 2:** Individual educational attainment prediction model performance.

	AUROC (95% CI)	Accuracy	F1-score	Precision
**Model 1: ZIP code-level Education**				
Naïve Bayes	0.64 (0.63 to 0.66)	0.55	0.26	0.25
Decision Tree	0.65 (0.64 to 0.66)	0.57	0.30	0.29
Random Forest	0.65 (0.64 to 0.66)	0.57	0.30	0.29
**Model 2: ZIP code-level Education + demographic data + use of tobacco, alcohol, and drug**				
Naïve Bayes	0.69 (0.68 to 0.71)	0.51	0.35	0.36
Decision Tree	0.71 (0.70 to 0.73)	0.62	0.37	0.30
Random Forest	0.75 (0.73 to 0.76)	0.61	0.31	0.30
**Model 3: ZIP code-level Education + demographic data + use of tobacco, alcohol, and drug + GINI and dissimilarity indices**				
Naïve Bayes	0.70 (0.68 to 0.71)	0.51	0.35	0.36
Decision Tree	0.74 (0.73 to 0.75)	0.62	**0.37**	**0.44**
Random Forest	**0.77 (0.76 to 0.78)**	**0.67**	0.31	0.32

### Performance of the ML model by race/ethnicity

The performance of the RF utilizing all features (Model 3) differed by patients’ race/ethnicity. The model performed best in the White group (AUROC 0.79), followed by the Asian group (AUROC 0.69), the Hispanic group (AUROC 0.68), and the Black group (AUROC 0.66), respectively **(S2 Table).**

The performance of all models improved when analyzed in White-predominant ZIP codes and lower in Black-predominant ZIP codes. Model 3 consistently demonstrated the best performance. Using RF in Model 3, the AUROC values in White-predominant and Black-predominant ZIP codes were 0.81 (0.79 to 0.82) and 0.72 (0.68 to 0.75), respectively, with corresponding accuracies of 0.71 and 0.56. **(S3 Table)**

### Cardiovascular disease hospitalization prediction

A total of 13,715 patients from the cohort had hospital visit records, with 1,538 (11.2%) of them having a history of CVD hospitalization. The mean age of the cohort was 54.2±15.4 years **(S4 Table).**

### Model performance

**Fig 2:**
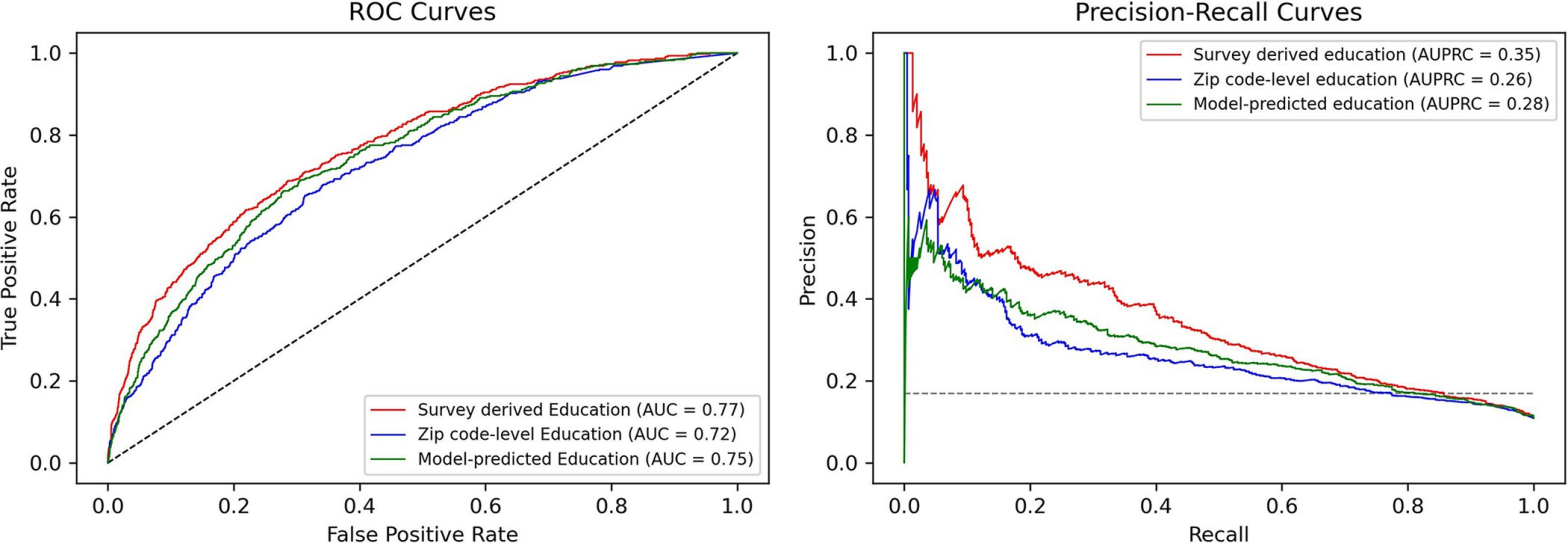
Receiver-operating characteristic and precision-recall curves for predicting CVD hospitalization of each model.

**Fig 3:**
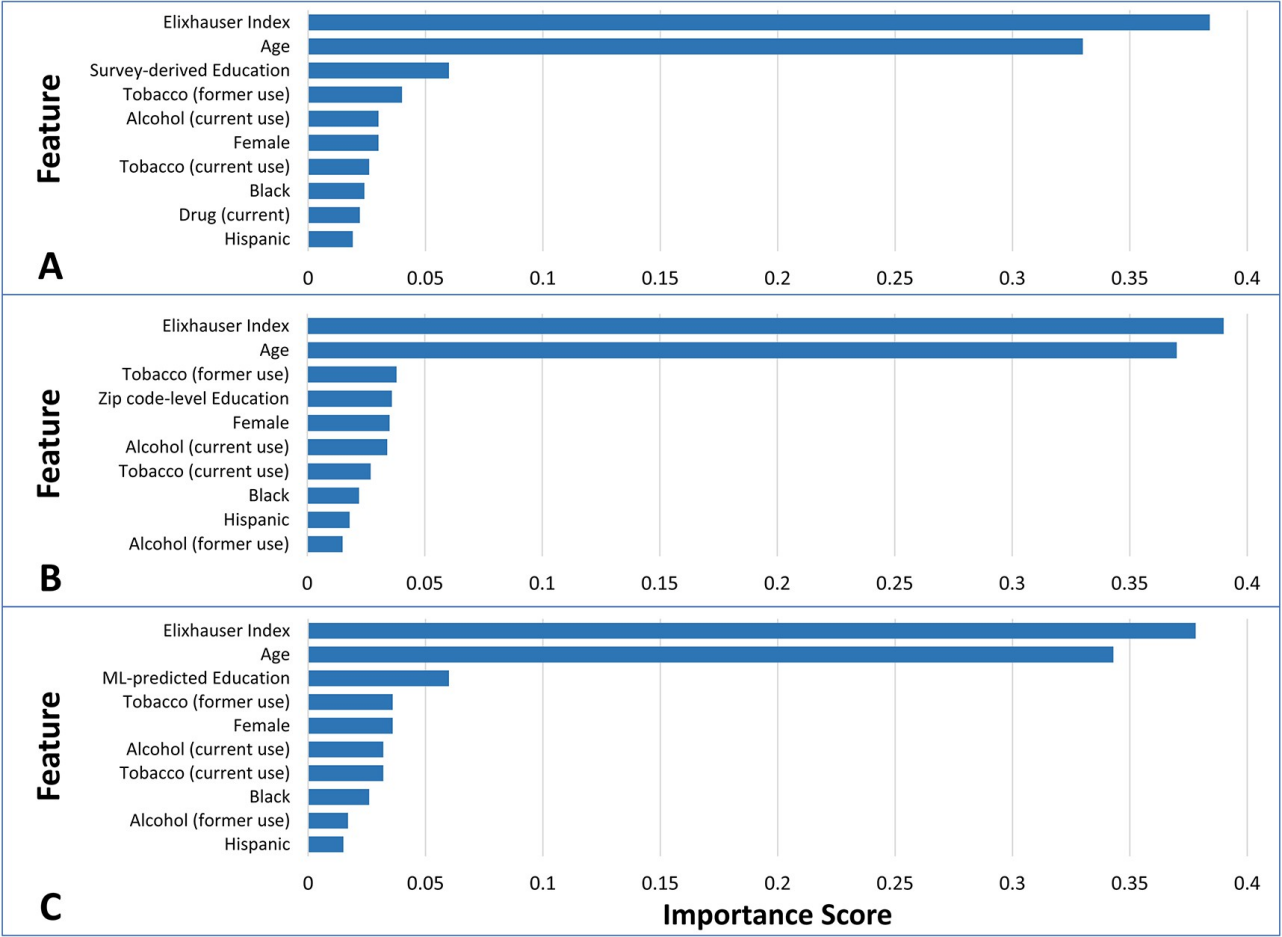
Feature importance ranking for CVD hospitalization prediction using RF algorithm. A. Survey-derived education model. B. Zip code-level education model. C. Machine learning-predicted education.

The performance metrics of all models for predicting CVD hospitalization are shown in Table 3. The model that did not include educational attainment as a variable achieved an AUROC of 0.72 and an AUPRC of 0.26. Incorporation of survey-derived education significantly improved the performance of the models. Among the models using different levels of education, the model based on survey-derived education demonstrated the highest accuracy and precision. Furthermore, it exhibited a significantly higher AUROC compared to the model using ZIP code-level education (0.77 versus 0.72; p < 0.001) and the model using ML model-predicted education (0.77 versus 0.75; p < 0.001). The AUROC for the model using the ML model-predicted education was also significantly higher than that of the model using ZIP code-level education (p = 0.003). Additionally, the model using survey-derived education achieved the highest AUPRC of 0.35, followed by the model using ML model-predicted education (0.28) and the model using ZIP code-level education (0.26). Fig 2 illustrate the ROC and PRC curves for each model. Fig 3 presents the rankings of the top 10 variables importance for predicting CVD hospitalization using the RF algorithm. The Elixhauser index was the feature that obtained the highest importance scores for prediction, followed by age, educational attainment, and former tobacco use, respectively.

**Table 3:** Predictive performance of CVD hospitalization models using different educational attainment methods.

Model	Model performance metric			
	Accuracy	Precision	AUPRC	AUROC
**All**				
Without education	0.87	0.48	0.26	0.71
Survey derived education	**0.89**	**0.55**	**0.35**	**0.77** *
ZIP code-level education	0.89	0.44	0.26	0.72
ML model-predicted education	0.89	0.51	0.28	0.75*
**White**				
Survey derived education	**0.90**	**0.67**	**0.35**	**0.77** *
ZIP code-level education	0.90	0.53	0.22	0.71
ML model-predicted education	0.90	0.67	0.28	0.75*
**Black**				
Survey derived education	**0.89**	**0.61**	**0.36**	**0.78** *
ZIP code-level education	0.87	0.44	0.30	0.73
ML model-predicted education	0.88	0.46	0.30	0.74
**Hispanic**				
Survey derived education	**0.90**	**0.88**	**0.36**	**0.76** *
ZIP code-level education	0.90	0.58	0.27	0.72
ML model-predicted education	0.90	0.60	0.30	0.75*
**Asian**				
Survey derived education	**0.89**	**0.48**	**0.46**	**0.83** *
ZIP code-level education	0.89	0.40	0.28	0.77
ML model-predicted education	0.89	0.38	0.28	0.78

* p-value < 0.05 compared with ZIP code-level education model

### Race/ethnicity-specific model performance

The model based on survey-derived education yielded the highest AUROC in all race/ethnicity groups. Among White and Hispanic patients, the models that utilized ML model-predicted education showed significantly higher AUROC values compared to those that were based on ZIP code-level education (p = 0.009 and p = 0.004, respectively). However, no significant differences in AUROC were observed within the Black and Asian groups (Table 3).

## Discussion

The findings in our study suggest a significant disagreement between education obtained via patient surveys compared to education derived from ZIP codes. Overall agreement was achieved in less than half of patients, and the level of agreement varied by gender, race, and ethnicity. We tested three ML algorithms and found that the RF model resulted in the best performance in the prediction of individual education. Additionally, we demonstrate that use of our ML-predicted educational attainment resulted in a significantly better CVD hospitalization model compared to models that used ZIP-code educational attainment. However, hospitalization models using survey educational attainment was significantly better than all the other models.

Previous studies have reported poor agreement between socioeconomic measures at the individual and area levels, as well as the impact of using these measures on the association between socioeconomic factors and health outcomes. Pardo-Crespo et al. [33] observed a lack of agreement between individual-level and area-level socioeconomic measures in Olmsted County, Minnesota, which is characterized by an urban-rural setting. The weighted Cohen’s κ indices ranged from 0.15 to 0.22. Furthermore, area-level socioeconomic measures revealed weaker associations with health outcomes in children, such as low birth weight and overweight, than individual-level socioeconomic measures. Moss et al. [34] found poor to moderate agreement between socioeconomic status, including household income, college degree, and unemployment, at individual, census tract, and county levels in the Mortality Disparities in American Communities (MDAC) study. When area-level measures were used as proxies for individual socioeconomic status, the associations between socioeconomic status and mortality were consistently underestimated.

NYC is a demographically and geographically diverse metropolis with approximately 214 ZCTAs designated by the U.S. Census Bureau across the five boroughs. The communities of NYC are characterized by racial/ethnic diversity and variation in the distribution of SDOH within their geographical boundaries. As a result, area-level SDOH measures may not accurately represent individual-level SDOH measures. Similar to prior studies, agreement between ZIP-code level education data with actual educational attainment in our study was low [33]. Therefore, we developed ML models to improve the prediction of individual-level education. By incorporating demographic data, racial segregation, and an income inequality index using the RF model, we achieved a noteworthy advancement in predicting individual educational attainment. This was demonstrated by a rise in the concordance rate to 67% and the AUROC to 0.77.

Additionally, we found that the concordance rate between individual and ZIP code-level education varied among races/ethnicities. Our ML models also performed differently across various race/ethnicity groups and significantly improved the concordance in Hispanics (from 31% to 52%) and Blacks (from 40% to 56%), groups that had the lowest concordance rate between survey and ZIP code-level education. To the best of our knowledge, this study represents the first attempt to apply area-level SDOH and multiclass ML algorithms for the prediction of individual SDOH.

Prior studies have examined whether the addition of SDOH into risk prediction models improves risk prediction accuracy. Individual SDOH measures have been reported as important features in CVD outcome prediction models [5, 35]. However, individual-level SDOH measures are difficult to obtain and often require direct patient surveys, which is time-consuming. Instead, many research studies use area-level SDOH measures as proxies; however, it is not clear if area-level SDOH improves risk prediction of clinical events.

We hypothesized that the inconsistency in the benefit of including neighborhood SDOH into prediction models of clinical outcomes is related to discordance of neighborhood and individual-level SDOH. Therefore, we evaluated the performance of a model to predict CVD hospitalizations using three different methods to identify educational attainment. Previous studies have reported a significant association between patient educational attainment and CVD hospitalization such as acute coronary syndrome and heart failure [19, 20]. As expected, survey-derived educational attainment had the best performance. While ZIP code-level education did not enhance the predictive performance of the CVD hospitalization model relative to the model without education, utilizing education data from our ML model significantly improved the prediction of CVD hospitalization. This improvement was particularly significant in Hispanic patients, where a low concordance rate was observed between survey and ZIP code-level education. However, we did not find a significant improvement in the model performance when using ML model-derived education in Black patients. These findings may be explained by the lower improvement of concordance between survey and ZIP code-level education by the ML model in the Black population.

Our study, to our knowledge, is the first to demonstrate and compare the impact of using SDOH derived from three different sources in predictive health outcome models. In addition, we have shown that ML-predicted SDOH measures not only improve individual-level SDOH prediction but also significantly boost the performance of a health outcome prediction model. Our study used a high-quality dataset with a large sample size and a racially and ethnically diverse cohort. The individual data utilized in our study was obtained from a self-reported questionnaire, and measurements of area-level education were derived from the ACS data for the same year. It is noteworthy that our participant pool encompassed over 85% of ZCTAs in NYC, providing comprehensive insights.

Our study placed particular emphasis on the importance of individual-level SDOH measures and raised concern regarding the utilization of area-level SDOH measures in public health research. To accurately assess individual SDOH data, it is crucial to employ well-designed and validated questionnaires with standardized data collection procedures. However, surveys are labor-intensive to obtain. Therefore, the use of models like ours may facilitate studies to assess the impact of SDOH on outcomes. In addition, the implementation of natural language processing (NLP) may enhance the extraction of SDOH information from clinical notes in EHR, thereby improving data collection. In studies where individual-level SDOH cannot be directly measured, caution should be exercised when interpreting associations between area-level SDOH proxies and health outcomes.

### Limitations

Several limitations should be acknowledged. Firstly, we utilized data from the Bio*Me* Biobank, which comprises individuals recruited from the Mount Sinai Health System, and the clinical outcomes were assessed in a single center, potentially limiting the generalizability. Secondly, although we aimed to include all participants in the Bio*Me* biobank, many participants did not complete the questionnaire and were excluded from the study, potentially leading to selection bias. Thirdly, we focused on education, which is only one specific aspect of SDOH. This limitation exists due to the lack of information in our database regarding additional SDOH measures that may be associated with education and health outcomes, such as household income, health insurance, and housing characteristics. While our results provide valuable insights, further studies are necessary to explore the extension of our findings to other SDOH measures. Additionally, examining their impact across various demographic subgroups and other health outcomes is warranted. Although our model improved the accuracy of educational attainment prediction for all racial/ethnic subgroups, its performance was still lower in Black and Hispanic individuals. Lastly, we did not conduct a full PRISMA literature review, and therefore may not include all relevant studies.

### Conclusions

The concordance of survey-derived and ZIP code-derived educational attainment in NYC was low. Our ML model significantly improved individual education prediction. In terms of CVD hospitalization prediction, the model utilizing survey-derived education achieved the highest performance. The model incorporating our ML model-predicted education outperformed the model relying on simple ZIP code-derived education. These findings suggest that the application of ML techniques has the potential to enhance the accuracy of SDOH data and consequently increase the predictive ability of CVD hospitalization prediction models.

## Supporting information

S1 Questionnaire (PDF)

S1 Fig CONSORT diagram. (TIF)

S1 Table Parameters in grid search. (DOCX)

S2 Table Individual educational attainment prediction model performance by race/ethnicity. (DOCX)

S3 Table Individual educational attainment prediction model performance by race/ethnicity-predominant ZIP codes. (DOCX)

S4 Table Patient characteristics for CVD hospitalization prediction. (DOCX)
