# Supplementary material for: Comparison of predicting cardiovascular disease hospitalization using individual, ZIP code-derived, and machine learning model-predicted educational attainment in New York City
Source: PLoS One. 2024 Feb 8;19(2):e0297919. doi: 10.1371/journal.pone.0297919 (PMC10852236; doi:10.1371/journal.pone.0297919)
Supplement: S1 Questionnaire — (PDF) [file pone.0297919.s001.pdf]

The Bio*Me* Biobank Program
Health Data Questionnaire

Patient ID Number: |_|_|_|_|_|_|_| **Date:** ___ /___ /___
MM / DD / YY

Height: |__|'|__||__|'' **Weight:** |__|__|__| lbs

Language preference (please indicate one): English____ Spanish____

Sex (please indicate one): Male____ Female____

A. ANCESTRAL HISTORY

1. Are you Hispanic/Latino?

No 0 ☐ → **GO TO QUESTION 2**
Yes 1 ☐ → **GO TO QUESTION 3**

2. Which of the following best describes your heritage? (You may select more than one)

American Indian, Native American or Alaskan Native	1 ☐
African-American / African	2 ☐
Caucasian / White	3 ☐
Mediterranean (for example from Spain, Portugal, Italy, Turkey, Greece, Middle East, and North Africa)	4 ☐
East or Southeast Asian (for example, from China, Japan, Korea, Indonesia)	5 ☐
South Asian/Indian (for example, from India, Pakistan)	6 ☐
Jewish	7 ☐
Native Hawaiian or Other Pacific Islander	8 ☐
Other:	

If other, please specify: ____________________________

3. Which of the following best describes your Hispanic/Latino heritage? (You may select more than one)

Dominican or Dominican descent	0 ☐
Central American or Central American descent	1 ☐
Cuban or Cuban descent	2 ☐
Mexican or Mexican descent	3 ☐
Puerto - Rican or Puerto Rican descent	4 ☐
South American or South American descent	5 ☐
Other	6 ☐

If other, please specify: ____________________________

4. In addition to being of Hispanic/Latino heritage, which of the following categories would you use to describe yourself? (Mark only one)

American Indian or Alaskan Native	1 ☐
Asian	2 ☐
Native Hawaiian or Other Pacific Islander	3 ☐
Black or African – American	4 ☐
White	5 ☐
Unknown or Not reported	6 ☐

B. EDUCATION

5. What was the highest grade/level of education achieved? If exact level is not listed, mark the closest equivalent. (Mark only one)

Elementary/primary school (includes grades 1 – 5)	1 ☐
Middle school/junior high (includes grades 6 – 8)	2 ☐
High School/preparatory school	3 ☐
Trade school/vocational school	4 ☐
University/college	5 ☐
Other	6 ☐

If other, please specify: _________________________

C. SMOKING

6.1 Have you ever smoked at least 100 cigarettes in your entire life?

No 0 ☐ → **GO TO QUESTION 7**
Yes 1 ☐

6.2 How old were you when you first started to smoke cigarettes fairly regularly?

__ __ Years old
☐ Never smoked cigarettes regularly

6.3 Do you NOW smoke daily, some days or not at all?

Daily	1 ☐ → GO TO QUESTION 7
Some days	2 ☐ → GO TO QUESTION 7
Not at all	3 ☐ → GO TO QUESTION 6.4

6.4. How old were you when you completely stopped smoking?

__ __ Years old

D. ALCOHOL USE

7.1. Do you presently drink alcoholic beverages?

No 0 ☐ → **GO TO QUESTION 7.3**
Yes 1 ☐

7.2. How often do you drink alcoholic beverages? (Mark only one)

Every day	1 ☐
5 to 6 days a week	2 ☐
3 to 4 days a week	3 ☐
2 days a week	4 ☐
1 day a week	5 ☐
2 to 3 days a month	6 ☐
1 day a month	7 ☐
Less than once a month	8 ☐
Never	9 ☐

7.3. Did you ever drink alcohol?

No 0 ☐
Yes 1 ☐

7.4. About how long ago did you stop drinking alcohol? (Mark only one)

Less than 1 year ago	1 ☐
1 - 2 years ago	2 ☐
More than 2 years ago	3 ☐
