# Supplementary figures and images for: Comparison of predicting cardiovascular disease hospitalization using individual, ZIP code-derived, and machine learning model-predicted educational attainment in New York City
Source: PLoS One. 2024 Feb 8;19(2):e0297919. doi: 10.1371/journal.pone.0297919 (PMC10852236; doi:10.1371/journal.pone.0297919)

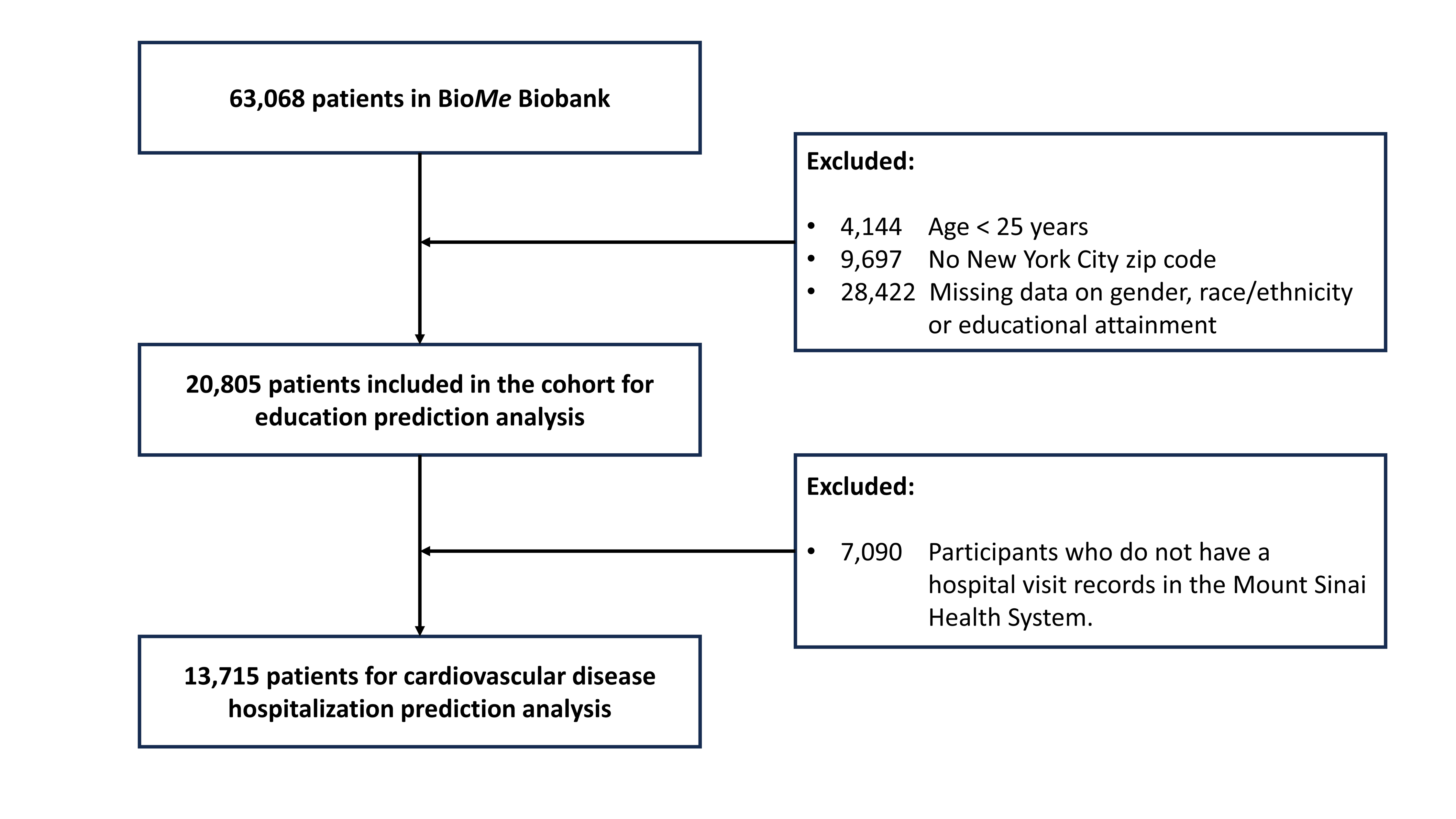

Supplement: S1 Fig — (TIF) [file pone.0297919.s002.tif]
