# Supplementary material for: Comparison of predicting cardiovascular disease hospitalization using individual, ZIP code-derived, and machine learning model-predicted educational attainment in New York City
Source: PLoS One. 2024 Feb 8;19(2):e0297919. doi: 10.1371/journal.pone.0297919 (PMC10852236; doi:10.1371/journal.pone.0297919)
Supplement: S1 Table — (DOCX) [file pone.0297919.s003.docx]

**S1 Table. Parameters in grid search**

| **Algorithm** | **Parameter grid** |
| --- | --- |
| **Decision tree** | param_grid = {'max_depth': range (1,10),  'min_samples_split': [2, 4, 6, 8, 10],  'min_samples_leaf': [1, 2, 3, 4, 5]} |
| **Random forest** | param_grid = {  'bootstrap': [True],  'max_depth': [2, 5, 10, 20],  'max_features': ['sqrt', 'auto'],  'min_samples_leaf': [1, 2, 3, 4, 5, 6, 7, 8],  'min_samples_split': [2, 4, 6, 8, 10],  'n_estimators': [500, 800, 1000, 1600, 2000]  } |
