# Supplementary material for: Comparison of predicting cardiovascular disease hospitalization using individual, ZIP code-derived, and machine learning model-predicted educational attainment in New York City
Source: PLoS One. 2024 Feb 8;19(2):e0297919. doi: 10.1371/journal.pone.0297919 (PMC10852236; doi:10.1371/journal.pone.0297919)
Supplement: S2 Table — (DOCX) [file pone.0297919.s004.docx]

**S2 Table.** **Individual Educational Attainment Prediction Model Performance by Race/Ethnicity**

|  | **AUROC (95% CI)** | **Accuracy** | **F1-score** | **Precision** |
| --- | --- | --- | --- | --- |
| **White (n = 5350)** |  |  |  |  |
| **Model 1: ZIP code-level Education** | | | | |
| Naïve Bayes | 0.53 (0.50 to 0.56) | 0.76 | 0.21 | 0.19 |
| Decision Tree | 0.53 (0.50 to 0.56) | 0.75 | 0.25 | 0.28 |
| Random Forest | 0.53 (0.50 to 0.55) | 0.75 | 0.25 | 0.28 |
| **Model 2: ZIP code-level Education + demographic data** | | | | |
| Naïve Bayes | 0.65 (0.62 to 0.67) | 0.64 | 0.28 | 0.29 |
| Decision Tree | 0.70 (0.68 to 0.73) | 0.76 | 0.25 | 0.31 |
| Random Forest | 0.75 (0.72 to 0.77) | 0.76 | 0.22 | 0.31 |
| **Model 3: ZIP code-level Education + demographic data + GINI and dissimilarity indices** | | | | |
| Naïve Bayes | 0.67 (0.64 to 0.69) | 0.64 | 0.28 | 0.29 |
| Decision Tree | 0.74 (0.72 to 0.76) | 0.76 | 0.29 | 0.31 |
| Random Forest | 0.79 (0.76 to 0.81) | 0.76 | 0.22 | 0.31 |
| **Black (n = 4446)** |  |  |  |  |
| **Model 1: ZIP code-level Education** | | | | |
| Naïve Bayes | 0.58 (0.55 to 0.61) | 0.46 | 0.17 | 0.24 |
| Decision Tree | 0.59 (0.56 to 0.62) | 0.54 | 0.28 | 0.27 |
| Random Forest | 0.59 (0.56 to 0.62) | 0.54 | 0.28 | 0.27 |
| **Model 2: ZIP code-level Education + demographic data + use of tobacco, alcohol, and drug** | | | | |
| Naïve Bayes | 0.62 (0.59 to 0.64) | 0.49 | 0.29 | 0.30 |
| Decision Tree | 0.59 (0.56 to 0.62) | 0.55 | 0.28 | 0.27 |
| Random Forest | 0.66 (0.63 to 0.69) | 0.55 | 0.28 | 0.28 |
| **Model 3: ZIP code-level Education + demographic data + use of tobacco, alcohol, and drug + GINI and dissimilarity indices** | | | | |
| Naïve Bayes | 0.62 (0.59 to 0.65) | 0.47 | 0.29 | 0.31 |
| Decision Tree | 0.59 (0.56 to 0.62) | 0.55 | 0.28 | 0.27 |
| Random Forest | 0.66 (0.63 to 0.68) | 0.56 | 0.28 | 0.28 |
| **Hispanic (n = 8858)** |  |  |  |  |
| **Model 1: ZIP code-level Education** | | | | |
| Naïve Bayes | 0.53 (0.51 to 0.55) | 0.42 | 0.23 | 0.21 |
| Decision Tree | 0.54 (0.52 to 0.56) | 0.46 | 0.23 | 0.23 |
| Random Forest | 0.54 (0.52 to 0.56) | 0.46 | 0.23 | 0.23 |
| **Model 2: ZIP code-level Education + demographic data + use of tobacco, alcohol, and drug** | | | | |
| Naïve Bayes | 0.63 (0.61 to 0.65) | 0.40 | 0.30 | 0.31 |
| Decision Tree | 0.64 (0.62 to 0.66) | 0.53 | 0.34 | 0.41 |
| Random Forest | 0.67 (0.65 to 0.69) | 0.52 | 0.29 | 0.27 |
| **Model 3: ZIP code-level Education + demographic data + use of tobacco, alcohol, and drug + GINI and dissimilarity indices** | | | | |
| Naïve Bayes | 0.63 (0.61 to 0.65) | 0.39 | 0.31 | 0.32 |
| Decision Tree | 0.61 (0.59 to 0.63) | 0.51 | 0.33 | 0.38 |
| Random Forest | 0.68 (0.66 to 0.70) | 0.52 | 0.29 | 0.26 |
| **Asian (n = 1121)** |  |  |  |  |
| **Model 1: ZIP code-level Education** | | | | |
| Naïve Bayes | 0.44 (0.38 to 0.49) | 0.76 | 0.23 | 0.23 |
| Decision Tree | 0.44 (0.38 to 0.49) | 0.76 | 0.24 | 0.23 |
| Random Forest | 0.44 (0.38 to 0.49) | 0.76 | 0.24 | 0.23 |
| **Model 2: ZIP code-level Education + demographic data + use of tobacco, alcohol, and drug** | | | | |
| Naïve Bayes | 0.72 (0.67 to 0.77) | 0.75 | 0.22 | 0.21 |
| Decision Tree | 0.60 (0.54 to 0.65) | 0.81 | 0.27 | 0.31 |
| Random Forest | 0.67 (0.62 to 0.73) | 0.82 | 0.22 | 0.20 |
| **Model 3: ZIP code-level Education + demographic data + use of tobacco, alcohol, and drug + GINI and dissimilarity indices** | | | | |
| Naïve Bayes | **0.71 (0.66 to 0.76)** | 0.76 | 0.22 | 0.21 |
| Decision Tree | 0.58 (0.52 to 0.63) | 0.80 | 0.24 | 0.25 |
| Random Forest | 0.69 (0.64 to 0.74) | 0.81 | 0.22 | 0.20 |
