# Supplementary material for: Comparison of predicting cardiovascular disease hospitalization using individual, ZIP code-derived, and machine learning model-predicted educational attainment in New York City
Source: PLoS One. 2024 Feb 8;19(2):e0297919. doi: 10.1371/journal.pone.0297919 (PMC10852236; doi:10.1371/journal.pone.0297919)
Supplement: S3 Table — (DOCX) [file pone.0297919.s005.docx]

**S3 Table. Individual Educational Attainment Prediction Model Performance by Race/Ethnicity-predominant ZIP Codes**

|  | **AUROC (95% CI)** | **Accuracy** | **F1-score** | **Precision** |
| --- | --- | --- | --- | --- |
| **White predominant ZIP codes (n = 7758)** |  |  |  |  |
| **Model 1: ZIP code-level Education** | | | | |
| Naïve Bayes | 0.62 (0.60 to 0.64) | 0.67 | 0.25 | 0.27 |
| Decision Tree | 0.61 (0.59 to 0.63) | 0.67 | 0.28 | 0.28 |
| Random Forest | 0.61 (0.59 to 0.63) | 0.67 | 0.28 | 0.28 |
| **Model 2: ZIP code-level Education + demographic data + use of tobacco, alcohol, and drug** | | | | |
| Naïve Bayes | 0.70 (0.68 to 0.72) | 0.60 | 0.36 | 0.36 |
| Decision Tree | 0.73 (0.71 to 0.75) | 0.72 | 0.38 | 0.44 |
| Random Forest | 0.79 (0.78 to 0.81) | 0.72 | 0.31 | 0.32 |
| **Model 3: ZIP code-level Education + demographic data + use of tobacco, alcohol, and drug + GINI and dissimilarity indices** | | | | |
| Naïve Bayes | 0.72 (0.70 to 0.74) | 0.60 | 0.35 | 0.36 |
| Decision Tree | 0.79 (0.77 to 0.80) | 0.70 | 0.37 | 0.42 |
| Random Forest | 0.81 (0.79 to 0.82) | 0.71 | 0.31 | 0.32 |
| **Black predominant ZIP codes (n = 2617)** |  |  |  |  |
| **Model 1: ZIP code-level Education** | | | | |
| Naïve Bayes | 0.57 (0.53 to 0.61) | 0.46 | 0.20 | 0.23 |
| Decision Tree | 0.56 (0.52 to 0.60) | 0.54 | 0.28 | 0.27 |
| Random Forest | 0.56 (0.52 to 0.60) | 0.54 | 0.28 | 0.27 |
| **Model 2: ZIP code-level Education + demographic data + use of tobacco, alcohol, and drug** | | | | |
| Naïve Bayes | 0.66 (0.62 to 0.69) | 0.49 | 0.34 | 0.35 |
| Decision Tree | 0.69 (0.65 to 0.73) | 0.59 | 0.33 | 0.38 |
| Random Forest | 0.72 (0.68 to 0.76) | 0.56 | 0.29 | 0.28 |
| **Model 3: ZIP code-level Education + demographic data + use of tobacco, alcohol, and drug + GINI and dissimilarity indices** | | | | |
| Naïve Bayes | 0.65 (0.62 to 0.69) | 0.47 | 0.34 | 0.34 |
| Decision Tree | 0.69 (0.65 to 0.73) | 0.56 | 0.31 | 0.34 |
| Random Forest | 0.72 (0.68 to 0.75) | 0.56 | 0.29 | 0.28 |
