# Supplementary material for: Comparison of predicting cardiovascular disease hospitalization using individual, ZIP code-derived, and machine learning model-predicted educational attainment in New York City
Source: PLoS One. 2024 Feb 8;19(2):e0297919. doi: 10.1371/journal.pone.0297919 (PMC10852236; doi:10.1371/journal.pone.0297919)
Supplement: S4 Table — (DOCX) [file pone.0297919.s006.docx]

**S4 Table. Patient Characteristics for CVD hospitalization prediction**

|  | **CVD hospitalization**  **(n = 1,538)** | **Non-CVD hospitalization**  **(n = 12,177)** | **p-value** |
| --- | --- | --- | --- |
| **Age** | 62.9 ± 13 | 53.1 ± 15 | < 0.001 |
| **Female** | 830 (54%) | 7434 (61%) | < 0.001 |
| **Race** |  |  | < 0.001 |
| Hispanic | 646 (42%) | 5639 (47%) |  |
| White | 307 (20%) | 2776 (23%) |  |
| Black | 491 (32%) | 2933 (24%) |  |
| Asian | 60 (4%) | 540 (4%) |  |
| Others | 33 (2%) | 289 (2%) |  |
| **Comorbidity** |  |  |  |
| Hypertension | 1144 (74%) | 5250 (43%) | < 0.001 |
| DM | 534 (35%) | 2164 (18%) | < 0.001 |
| CKD | 498 (32%) | 1245 (10%) | < 0.001 |
| **Elixhauser index** | 6 (4-8) | 3 (1-5) | < 0.001 |
| **Tobacco use** |  |  |  |
| Current | 402 (26%) | 2705 (22%) | < 0.001 |
| Former | 807 (52%) | 4475 (37%) | < 0.001 |
| **Alcohol use** |  |  |  |
| Current | 645 (42%) | 6196 (51%) | < 0.001 |
| Former | 111 (7%) | 635 (5%) | 0.001 |
| **Drug use** |  |  |  |
| Current | 238 (15%) | 1679 (14%) | 0.08 |
| Former | 63 (4%) | 327 (3%) | 0.002 |
| **Survey-derived educational attainment** |  |  | < 0.001 |
| Less than a high school diploma | 219 (14%) | 1225 (10%) |  |
| High school graduate | 732 (48%) | 4898 (40%) |  |
| Some college or associate degree | 9 (1%) | 127 (1%) |  |
| Bachelor’s degree or higher | 578 (37%) | 5927 (49%) |  |
